# A radiological index that influences the outcome following patellofemoral joint arthroplasty: the anterior trochlea offset ratio

**DOI:** 10.1007/s00167-022-07085-1

**Published:** 2022-09-13

**Authors:** Osama Aweid, Nathanael Ahearn, Andrew J. Metcalfe, Jonathan Eldridge, Andrew Porteous, James R. Murray, The Bristol Knee Group, Andrew Porteous, Jonathon Eldridge, James Robinson, James Murray, Hywel Davies, Nick Howells, Damian Clark, Sven Putnis, Mo Hassaballa, Rachel Bray, Corina Negrut, Karen MacDonald, Suzanne Miller

**Affiliations:** Avon Orthopaedic Centre, Southmead Hospital, Bristol, BS10 5NB UK

**Keywords:** Patellofemoral joint arthroplasty, Knee, Functional outcome, Component position, Radiological review

## Abstract

**Purpose:**

Although largely successful, patellofemoral joint arthroplasty (PFA) has a less than satisfactory outcome in some patients. It was hypothesized that certain factors can be identified on radiological review that correlate with poor patient reported outcomes following PFA.

**Methods:**

A retrospective cohort review of 369 patients undergoing PFA at our institution between 2005 and 2018 identified 43 “poor outcome” patients with an Oxford Knee Score (OKS) of less than 20 at 2 years follow up. These cases were matched by sex and age with 43 “good outcome” patients who had an OKS above 40 at 2 years post-op. Multiple radiological measurements were performed including anterior trochlea offset ratio (ATOR), component flexion/extension, component varus/valgus, component to bone width ratio and retinacular index. The OKS PROM was the primary outcome of the study. Stepwise logistic regression was performed to analyze the differences in radiological indices between the two groups.

**Results:**

Intraclass correlation coefficients for inter-observer and intra-observer reliability were 0.90–0.98 for all indices measured. The only index demonstrating statistical significance between the groups was the ATOR (*p* = 0.003). The good outcome group had a mean ATOR of 0.19 whereas the poor outcome group had a mean ATOR of 0.24.

**Conclusions:**

Lower ATOR on radiological review was strongly associated with improved outcomes following PFA. The surgeon should therefore take particular care to prevent increasing the anterior offset of the trochlea component when performing PFA.

**Level of evidence:**

Retrospective cohort study, Level III.

**Supplementary Information:**

The online version contains supplementary material available at 10.1007/s00167-022-07085-1.

## Introduction

Although patellofemoral joint arthroplasty (PFA) is largely successful, there remains a group of patients who experience poor functional outcome following surgery. This is likely related to either progression of tibiofemoral arthritis, or alteration of PFJ biomechanics resulting in persistent PFJ pain and dysfunction [3].

Implant positioning during PFA can affect PFJ biomechanics however the relationship between measurements of the former and clinical symptoms or functional outcome is yet to be established. ‘Overstuffing’ of the PFJ has not been clearly defined in the literature, although it has been proposed to increase patella-related complications following TKA [8, 13]. A number of papers have attempted to describe PFJ 'overstuffing' and have reported measures of anterior offset [13, 14] or patella thickness [17] on radiographs and tried to relate these to outcome, without success. These measures could be criticized, however, for lacking any method of normalizing the measurements to the patient’s size or scaling of the radiographs, resulting in an underestimation of the true effects of component mal-position. Furthermore, the effect of other radiological parameters on outcome following PFA, such as varus/valgus, flexion/extension, and the implant width, have not been confirmed. Identifying such factors would be invaluable as they would direct the surgeon as to what the optimal implant positioning should be intra-operatively. Post-operatively there is also the potential for retrospective application of any proven index in the assessment of painful PFAs.

The aims of this study were to develop one or more methods of quantifying the radiographic positioning of a PFA referenced against individual patient anatomy, and to determine if abnormalities in these measures are related to functional outcome. It was hypothesized that higher radiological anterior offset of the trochlea component correlates with poor outcome post-operatively given that it indicates an increase in the overall height of the patellofemoral construct and therefore “overstuffing”.

## Materials and methods

The STROBE guidelines were used to ensure the reporting of this observational study (see additional file 1). Ethical approval was obtained for this study from the Clinical Governance and Audit department at North Bristol NHS Trust, UK”. All patients undergoing PFA in our unit are recorded prospectively on the Bristol Knee Group (BKG) database, and a retrospective case–control analysis was performed on this database. Both the Avon (Stryker Orthopaedics, Mahwah, New Jersey) and Journey (Smith and Nephew, Memphis, Tennessee) PFA are used in our unit and were included in this study although Avon use favoured the earlier years when the Journey PFJ was not available. Patients undergo routine clinical and radiological review preoperatively, and at regular intervals postoperatively, where clinical measures (range of motion, complications, further operations) and patient reported outcome measures (PROMS) are collected. The Oxford Knee Score (OKS) PROM was the primary outcome of the study.

The BKG database at the time of this study contained 1,087 implants (857 Avon, 130 Journey PFA). Cases over fifteen years old were excluded to ensure radiographs were available on the electronic image archiving system. Cases without two year post-operative follow up were also excluded leaving a total of 369 implants (254 Avon, 115 Journey PFA).

**Fig. 1:**
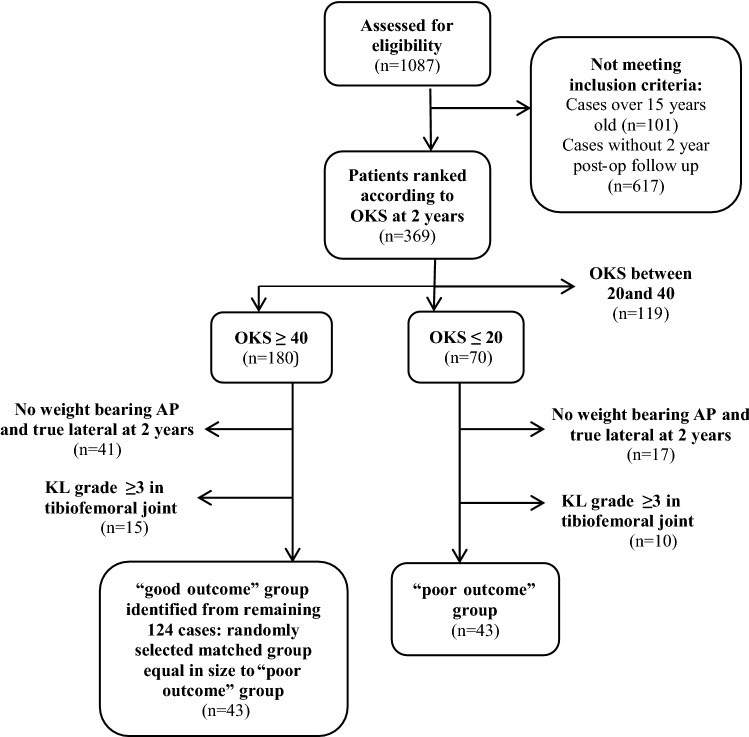
Flow chart of patient recruitment and data collection

Patients were ranked according to OKS at two years postoperatively, as this has previously been shown for TKA to be the time at which the best OKS should be expected [15]. The OKS ran from 0 (worst) to 48 (best). Patients with an OKS ≤ 20 were identified from each implant and defined as the 'poor outcome' group. Patients without a weightbearing antero-posterior and a true lateral radiograph of the knee at 2 years postoperatively were excluded. To prevent cases of early tibiofemoral disease progression confounding the findings of our study, patients with a Kellgren–Lawrence grade ≥ 3 in the tibiofemoral compartment were also excluded. This left a total of 43 with an OKS of less than 20 (see Fig. 1). For the good outcome” group, 124 cases were identified after applying the same inclusion and exclusion criteria. For each poor outcome patient a “good outcome” case was randomly selected out of the 124 identified, through an Excel generated list of random numbers, and matched by sex and age (± 5 years of the poor outcome case’s age). The inclusion and exclusion criteria are summarized in Table 1. The OKS was selected at these levels based upon previous reports of patient satisfaction following hip or knee arthroplasty surgery. Scores below 20 have been correlated with poor satisfaction [9] whereas those above 37–42 had higher satisfaction and willingness to undergo surgery again [10].

**Table 1:** Inclusion and exclusion criteria

Inclusion criteria	Exclusion criteria
Patients undergoing either an Avon or Journey PFA	Cases performed over 15 ago
Cases performed between 2005 and 2018	No weight bearing AP and true lateral at 2 years
Cases with a 2 year follow up	KL grade ≥ 3 in tibiofemoral joint

Radiological measurements were performed by two independent raters (authors NA and AM) electronically using Synapse (Fujifilm UK Limited, Bedford, UK) picture archiving system on a total of 86 PFA (43 ‘good outcome' and 43 ‘poor outcome’) undertaken at our institution between 2005 and 2018. A repeatability study was performed on each of these measurements, using ten PFA implants (five Avon and five Journey) with two observers (NA and AM), each recording twice at a time interval of seven days. The order of measuring was changed between the different time occasions, using blinded radiographs, and the values recorded were used to determine intra- and inter-observer variability.

Three measurement techniques were assessed on lateral radiographs of the knee, the ATOR, the flexion/extension of the implant, and the retinacular index.

**Fig. 2:**
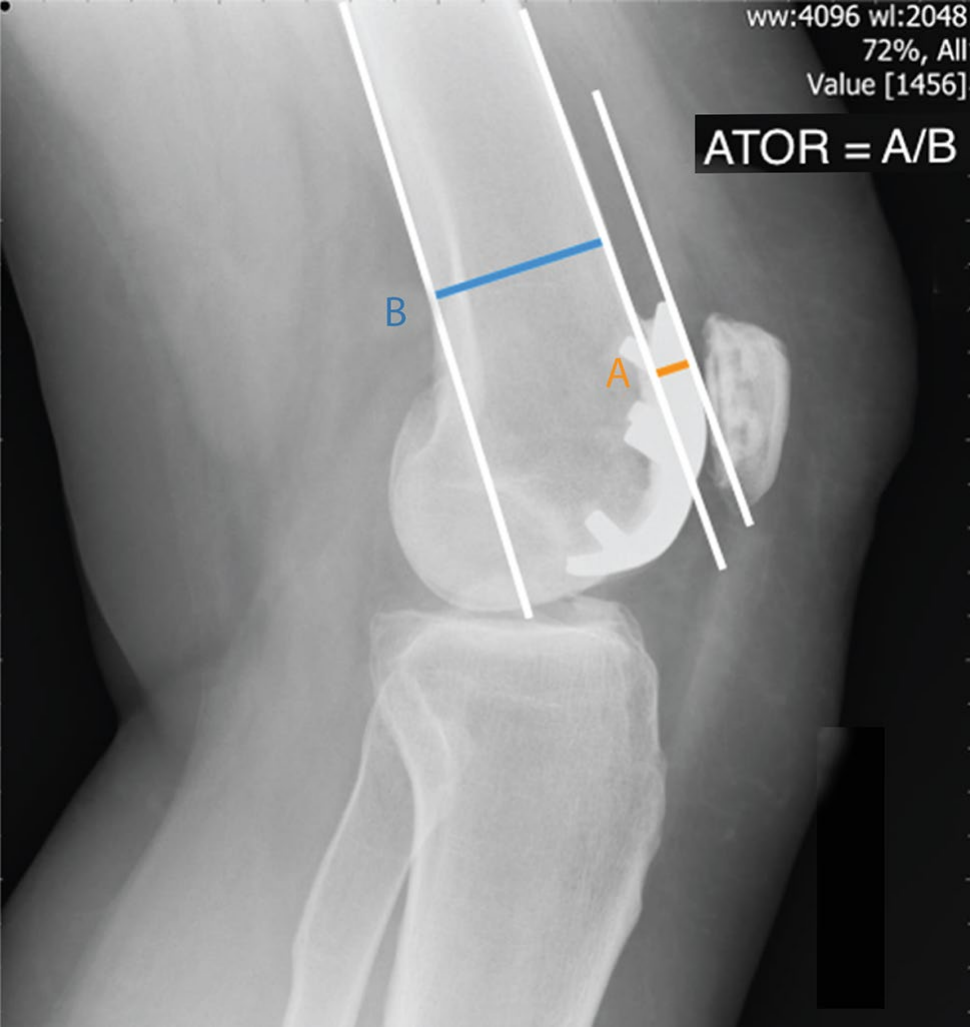
Measurement of component anterior trochlea offset ratio (ATOR)

**Fig. 3:**
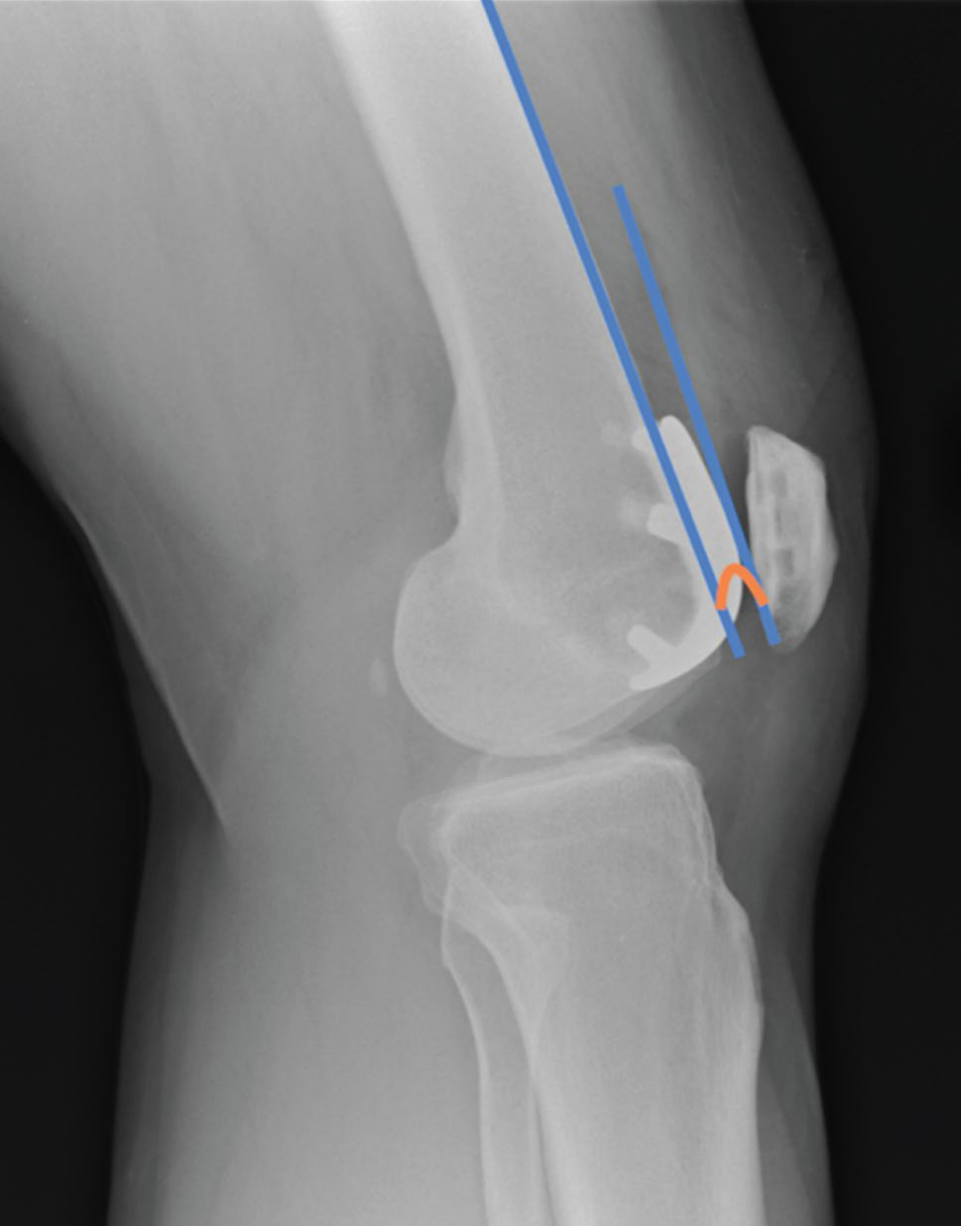
Measurement of component flexion/extension

The ATOR is analogous to the posterior condylar offset ratio [5], but for the anterior compartment. The ATOR is a new ratio that we have defined to measure anterior implant positioning but controlling for variation in patient size whilst describing the anterior trochlea height relative to the bone width on lateral radiograph. It was calculated by dividing the Antero-posterior width of the trochlear component by the antero-posterior width of the femoral shaft (Fig. 2). The flexion/extension of the component was measured as the angle between the posterior surface of the anterior flange of the trochlea component and the anterior femoral shaft (Fig. 3). A positive value signified the implant was in flexion, with a negative value signifying the implant was in extension.

**Fig. 4:**
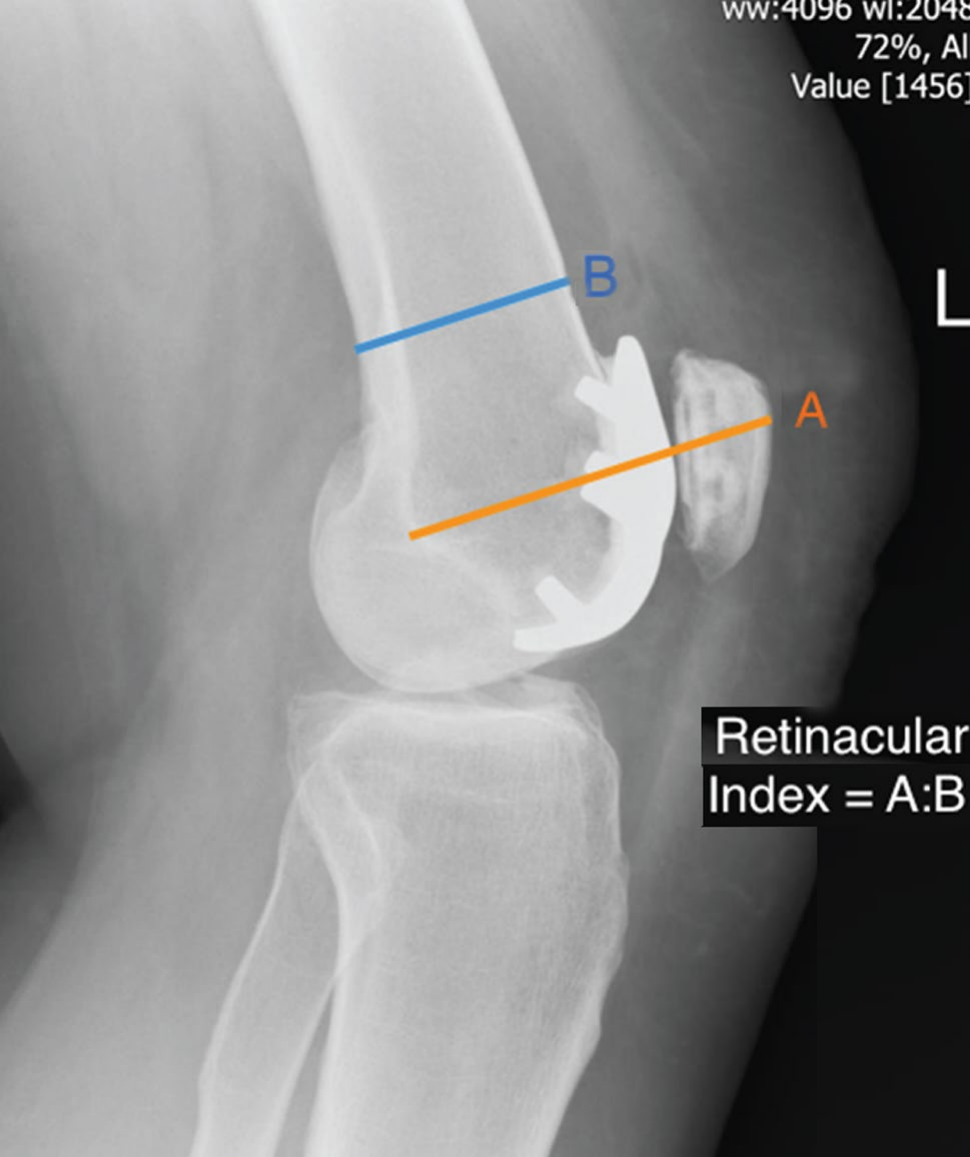
Measurement of component retinacular index

**Fig. 5:**
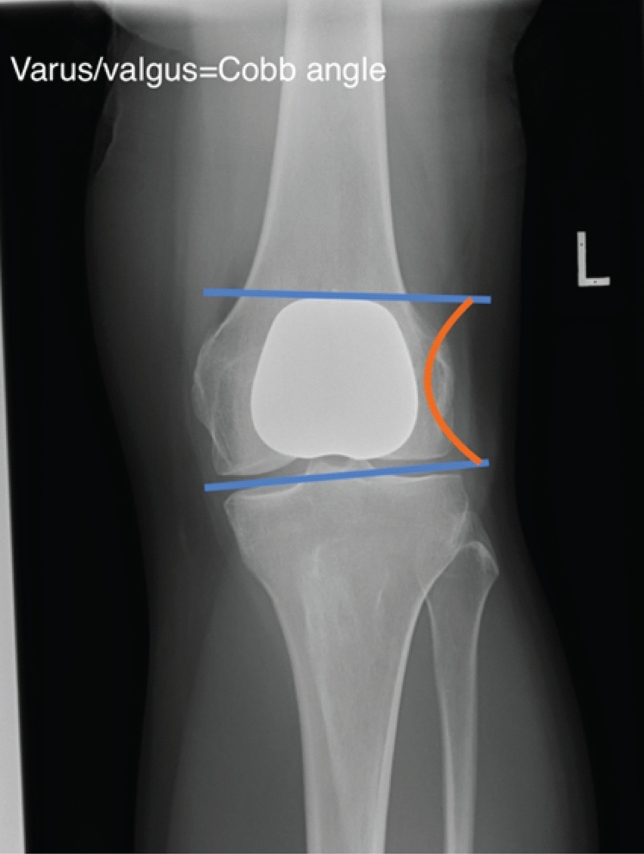
Measurement of component varus/valgus

**Fig. 6:**
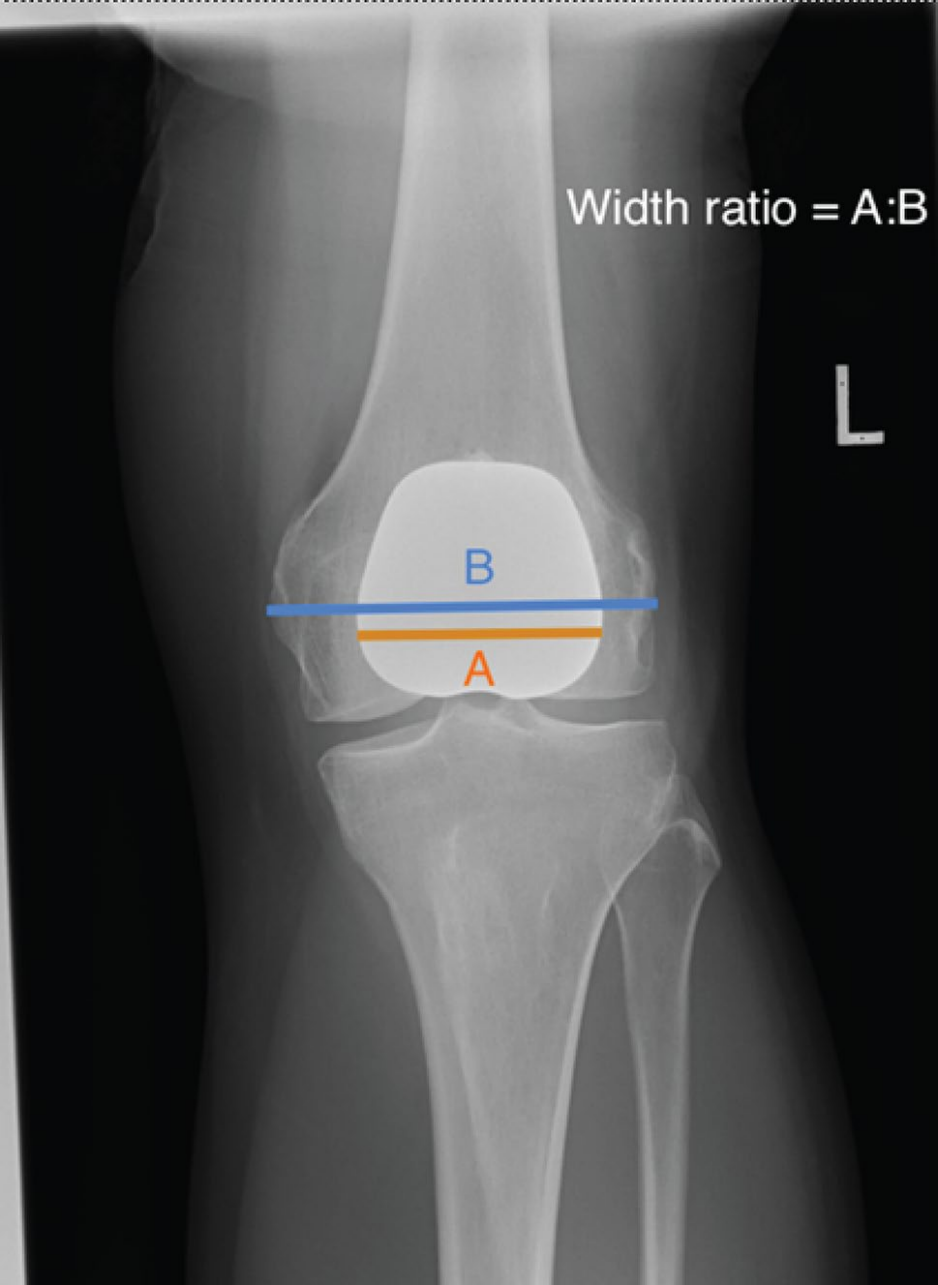
Measurement of component width ratio

We devised the “retinacular index” to define PFJ ‘overstuffing’ by determining the overall height of the patellofemoral construct. As shown in Fig. 4, this was calculated by dividing line A (the distance between the posterior end of Blumensaat’s line which is in close proximity to the insertion of the medial patellofemoral ligament [1], to the midpoint of the patella button) by line B (the antero-posterior width of the femoral shaft). On the antero-posterior radiographs the varus/valgus (Fig. 5) of the implant was measured as the angle subtended between the femoral joint line and the superior border of the trochlea implant). The implant to femur width ratio was measured by dividing the widest part of the implant by the distance between the femoral epicondyles (Fig. 6).

It was initially intended that patella tilt would be measured, but skyline radiographs were not found to be sufficiently reliable to allow this measure to be made in all cases, so it was discarded.

### Statistical analysis

The statistical analysis was performed using a stepwise logistic regression, with the ATOR, flexion/extension, retinacular index, varus/valgus, and width ratio as co-factors. An analysis was made of the agreement of the radiological reviewers using the intraclass correlation coefficient (ICC) as defined by Shrout and Fleiss [16]. ICC values less than 0.5, between 0.5 and 0.75, between 0.75 and 0.9, and greater than 0.90 were used to indicate poor, moderate, good, and excellent reliability, respectively [12].

The differences between the ‘good outcome’ and ‘poor outcome’ PFA group characteristics were evaluated using stepwise logistic regression analysis. A *p* value < 0.05 was considered to be statistically significant. No formal pre-intervention power calculation was performed. However, a post hoc power calculation for the sample size used revealed an actual power of 84% for finding the observed difference in mean ATOR between the “good outcome” and “bad outcome” group. All statistical analyses were performed using SPSS 21 (IBM Corp, Armonk, New York).

## Results

The mean age was 59 years (29–88), with PFA implanted in a total of 65 females and 21 males. The mean change in score at two years postoperatively was 19.12 points higher in the ‘good outcome’ group compared to the ‘poor outcome’ group (*p* < 0.001, see Table 2).

**Table 2:** Patient characteristics of the 'good' and 'poor' outcome groups

	Good outcome (*n* = 43)	Poor outcome (*n* = 43)	*p* value
Avon: Journey	22: 21	26: 17	0.39
Age (years)	62.1 (16.7)	56.6 (14.3)	0.10
Sex	12 M: 31F	9 M: 34F	0.65
Mean OKS at 2 years	44.7 (2.48)	12.5 (4.05)	* < 0.001
Mean OKS change	21.68 (4.67)	2.56 (4.67)	* < 0.001

Continuous data are presented as mean ± standard deviation (SD). Avon:Journey = the number of Avon and Journey PFA respectively. *p < 0.05

Intraclass correlation coefficients for inter-observer and intra-observer reliability were 0.90–0.98 for all indices measured. The logistic regression identified ATOR as the only radiological index achieving statistical significance that correlated with outcome (*p* = 0.003) (Table 3). The poor outcome group also had a higher mean width ratio and retinacular index, however, the difference did not reach statistical significance. The mean flexion/extension and varus/valgus index was lower in the poor outcome group but the difference did not reach statistical significance as well.

**Table 3:** Absolute mean values and *p* values for radiological indices measured [(± SD), *p < 0.05)]

Radiological Index	Good outcome mean	Poor outcome mean	*p* value
ATOR Anterior trochlea offset ratio	0.19 (0.091)	0.24 (0.081)	0.003*
Width ratio	0.58 (0.037)	0.60 (0.041)	0.140
Retinacular Index	2.07 (0.21)	2.15 (0.16)	0.480
Flexion/extension	5.16° flexion (6.52°)	3.67° flexion (6.85°)	0.551
Varus/valgus	5.42° valgus (3.68°)	5.26° valgus (4.15°)	0.883

## Discussion

The main finding of this study was that higher implant ATOR on post-operative plain films (0.24 vs 0.19) is associated worse clinical outcomes at 2 years following PFA. This measure also showed good reliability with inter-observer and intra-observer correlation coefficients of 0.90–0.98. The other measurements of implant position, component flexion/extension, varus/valgus, retinacular index and width ratio, did not show a statistically significant association with outcomes.

Isolated patellofemoral joint (PFJ) osteoarthritis can be successfully managed with PFA using a variety of implants. A recent randomized controlled trial by Joseph et al. [6] of 64 patients with severe isolated patellofemoral arthritis found similar functional outcome at 12 months and mid-term in the use of PFA compared with total knee arthroplasty (TKA). Odgaard et al. [15] in their 2-year results of a multicentre randomized clinical trial with 50 patients per arm, reported similar 36-Item Short-Form Health Survey questionnaire and bodily pain scores at 1- and 2-year follow-up, however, early postoperative patient reported outcome scores favoured PFA. Knee Injury and Osteoarthritis Outcome Score also demonstrated a difference of 10 points between the groups at two years in favour of PFA (*p* = 0.023).

The reasons for poor outcome following PFA are often complex and multi-factorial, but can be broadly divided into surgical and non-surgical causes. Implant position is possibly the key surgical factor involved. Trochlear-cutting PFA improves patellofemoral congruence by correcting trochlear dysplasia and standardising radiological measurements [18]. Poor implant position might be expected to worsen pain in a patient who is already pre-disposed to chronic postsurgical pain, or it may be an independent factor. Mofidi et al. [14] in their series found, however, no difference in outcome by increasing anterior implant height (in millimetres) following PFA using the FPV implant although their series had smaller numbers (34 total PFA) and a shorter follow-up (6 months) than ours. Matz et al. [13] in their large series reporting outcomes following TKA also concluded that PFJ over-stuffing does not lead to adverse outcomes. Kandhari et al. described a patella-femoral composite (PFC) distance, the maximum distance between the anterior cortical line of femur and the anterior cortex of patella, and were able to correlate this with postoperative passive knee range of motion but not function or pain [7].

The findings above are in contrast to the present study where a measure of PFJ over-stuffing, the ATOR, was correlated with post-operative function and pain. This may be due to the above studies utilizing quantitative measure in millimetres of anterior height as opposed to a ratio which controls for patient size. Surprisingly, the retinacular index, a composite ratio designed to assess for true patellofemoral overstuffing, was not associated with postoperative outcomes. This may be due to the possible variability in this measurement depending on the degree of knee flexion when the radiograph was taken. It is also possible that patella thickness has no association with true overstuffing and less important clinically as suggested by some studies [4, 11]. The ATOR in contrast, does not vary with knee flexion. Its relevance to patellofemoral overstuffing and clinical outcomes is further supported by evidence that TKA designs with an in-built increase in trochlea height of the femoral component have been shown to increase the need for secondary patellar resurfacing [19].

Our results support the findings of Kemp et al. who associated an increase in ATOR following a Triathlon^®^ (Stryker, Kalamazoo, MI, USA) TKA with worse knee pain and function scores. Very large changes in ATOR (three times the preoperative value) were required, however, (three times the preoperative value) to cause clinically important changes in postoperative WOMAC score [8]. In practice this would only be possible in patients with a very low preoperative ATOR suggesting a less important role for ATOR in TKA compared to PFA. This is possibly because the factors influencing outcome following TKA are more variable than those affecting isolated PFA, with the whole femoral condyle resurfaced and no reliance on the native knee ligaments to control knee function following surgery. The inherent nature of TKA will adversely alter knee joint kinematics, whereas the aim of PFJ is to optimise PFJ kinematics and thus the implants are more prone to problems created by subtle component malalignment.

The association between ATOR and post-operative patient outcomes has important clinical implications. With this knowledge, orthopaedic surgeons could take extra care ensuring their distal femoral resection does not inadvertently increase the ATOR. This can be achieved by firstly ensuring an adequate amount of bone is removed anteriorly to allow the trochlea component to sit flush or slightly deep to the adjacent native joint surface. Careful attention to femoral rotation will also avoid maltracking or an increase in ATOR by avoiding excess elevation of one side of the trochlear component. Similarly, consideration of implant flexion/extension would avoid elevation of the anterior trochlear offset proximally or distally. Measuring ATOR could also be of use in the assessment of painful PFAs with values above 0.19 suggestive of trochlea positioning as a possible cause. In terms of external validity, the results of this study can be generalized to other populations as the age, gender and implant brand distribution in this cohort reflect that in populations reported in national registries.

**Fig. 7:**
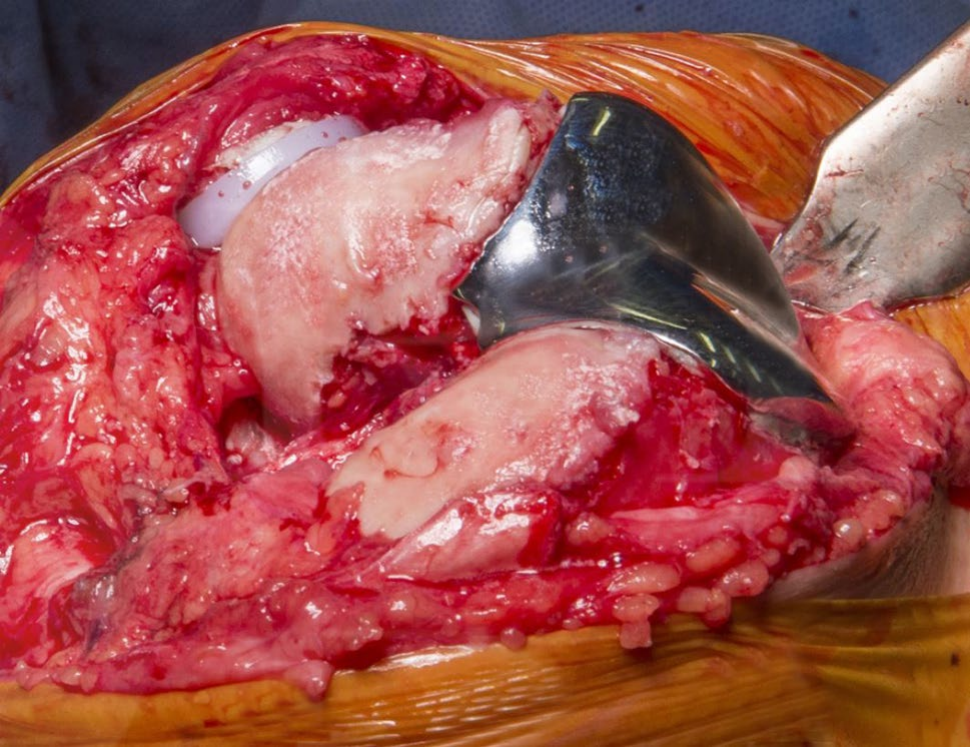
A clinical photograph of the Avon Patellofemoral joint Arthroplasty (PFA)

Our study has limitations. This is a retrospective study and has inherent bias associated with this retrospective study design. The numbers are relatively small, although large studies of PFA are rare in the literature as PFA accounts for around 1% of knee arthroplasty. Moreover a post-hoc power analysis revealed that the study was sufficiently powered for finding the observed difference in mean ATOR between the “good outcome” and “bad outcome” group. A further limitation is that both implants were combined in the analyses despite having different designs. The Avon is very wide proximally compared to the Journey and has the same design for both left and right knees (see Fig. 7).

The study has shown a potential radiological marker of poor outcome following PFA in two implants used, but further work is needed to determine if the results are also applicable to other PFA or TKA implants. As a purely radiological study there was no measure of other clinical factors that may affect outcome. There may also be other non-surgical factors, such as patient age and Body Mass Index (BMI), that may have a greater effect on patient reported outcome measures following PFA than radiological indices alone. The ‘poor outcome’ group had a lower mean age by 5.5 years and the effect of patient age, with lower scores in younger individuals, has been noted for TKA previously [2]. The comprehensive analysis of patient and pre-morbid factors would require a very large cohort of patients that is beyond the scope of this current study. The use of cross-sectional imaging such as CT scans could potentially have provided further information, particularly with regards to rotation of components, which can be difficult to assess on plain radiographs alone. The use of CT scans, however, is not routinely performed in our clinical practice unless we are investigating problematic knee arthroplasty.

## Conclusions

In conclusion, higher implant ATOR on post-operative plain films is associated with worse clinical outcomes at 2 years following PFA. There is no association, however, between clinical outcomes and implant flexion/extension, varus/valgus, retinacular index or width ratio in this study. The surgeon should therefore take particular care to prevent increasing the anterior offset of the trochlea component during PFA surgery.

## Supplementary Information

Below is the link to the electronic supplementary material.

Supplementary file1 (DOCX 26 KB)

## Abbreviations

ATOR: Anterior Trochlea Offset Ratio
PFA: Patellofemoral joint Arthroplasty
TKA: Total knee arthroplasty
PFC: Patella-femoral composite
BKG: Bristol Knee Group
OKS: Oxford Knee Score
ICC: Intraclass correlation coefficient
BIPWiT: Bristol index of patellar width to thickness
